# Identification and Characterization of Bluetongue Virus Serotype 14 in Russia

**DOI:** 10.3389/fvets.2020.00026

**Published:** 2020-02-28

**Authors:** Andrei Koltsov, Sodnom Tsybanov, Andrey Gogin, Denis Kolbasov, Galina Koltsova

**Affiliations:** Federal Research Center for Virology and Microbiology, Pokrov, Russia

**Keywords:** bluetongue virus, *Orbivirus*, phylogenetic analysis, serotype 14, blood-sucking midge vectors, spread, Russia

## Abstract

This paper reports a case of bluetongue virus (BTV) infection in the Smolensk and Kaluga regions of Russia in 2011–2012. The virus was initially detected in heifers transferred in Russia from Germany through Poland and Belarus in 2011. On day 27 of quarantine, RNA and infectious viruses of BTV were detected in four heifers, but five were serologically positive. However, on day 3 before shipment, all heifers were seronegative and PCR-negative for BTV. Thus, a few animals from this consignment were viremic without any evident subclinical infection. Based on Seg-2 (VP2 gene) and Seg-5 (NS1 gene) sequencing, the recovered virus had 99.86–100% nucleotide identity with BTV-14-like viruses such as the vaccine BTV-14 strain RSArrrr/BTV 14 and the BTV-14 isolates detected in Lithuania and Poland in 2012. Subsequently, BTV-14 was also reported in local animals in two regions of Russia. During the monitoring survey, 1623 local animals within a 300-km radius were tested, of which 471 tested positive by ELISA and 183 by PCR for BTV-14 RNA. No other serotypes were identified in either imported or aboriginal animals within that radius. The *Culicoides* midges trapped at the site of the outbreak in May 2012 tested positive for the BTV-14 genome, indicating that the possible mechanism of spread most likely occurs via vector bites. However, further investigation is required to confirm this hypothesis, which would provide an improved understanding of the circulation and overwintering of BTV in northern latitudes.

## Introduction

Historically, bluetongue (BT) was considered endemic within Africa and was first discovered after fine-wool sheep imported from Europe (1). The affected sheep exhibited the typical symptoms of swollen lips and tongue accompanied by a characteristic tongue cyanosis, giving a name to the disease (2).

The etiologic agent of BT was later discovered to be a double-stranded RNA virus with a segmented genome of the genus *Orbivirus* and family *Reoviridae* (3, 4). The virus is transmitted by blood-sucking midges of the genus *Culicoides* (5–8). Ticks have also been implicated as a vector (9). A total of 27 serotypes were recently recognized, with another few pending (10–16). The serotypes exhibit various patterns of transmission, including direct contact (17, 18), which contradicts the accepted concept that bluetongue virus (BTV) is only transferred via a *Culicoides* vector (19).

BTV is endemic throughout Africa within the range of its primary vector *Culicoides imicola*. The virus was first confirmed outside Africa in India (20) and then in Australia, where the virus was isolated from *Culicoides* collected near Darwin in 1975 (21). The distribution of BTV has been substantially expanding, although historically, before 1998, its range was confined to regions between the latitudes of 35°S and 40°N (7).

BTV was never expected to reach areas far beyond its traditional northernmost latitude. That could be explained by the relatively cold climate and short period of seasonal midge activity preventing this virus from establishment [reviewed by (22)]. However, in 2008, BT arrived in northern Europe and caused unprecedented outbreaks (7). The arrival of BTV could be attributed to the movement of infected ruminants or the wind dispersal of infected midges (23). Notably, the key role in the transmission of BTV in northern Europe was played by novel Palaearctic vectors, the Obsoletus and Pulicaris complexes (24). Considering the abundance of vectors and the recent changes in climate, it is not surprising that new BTV strains have appeared. These strains include reassortants and serotypes from the Mediterranean region, which are now considered endemic, and new strains are expected to continue to regularly appear (18, 25, 26), putting Eastern Europe and the European part of Russia at risk.

In Russia, the first historical outbreak of BTV among sheep caused morbidity of 58.3% and mortality of 66.3% in 1993 in Eastern Siberia (Republic of Buryatia) (27). Vaccination successfully contained the outbreak. The serotype was identified as BTV16, which is related to the eastern topotype (unpublished data). Considering that Europe has recently experienced multiple incursions of different serotypes (25), the risks of BTV introduction into European Russia has increased dramatically owing to cattle trading between Russia and Europe. The first cases of BTV-8 infection were identified in cattle imported from Germany and the Netherlands in 2008 (28). Notably, BTV-8 was only isolated from imported animals. BTV-8 infection among local animals was prevented as all PCR and seropositive animals were immediately slaughtered (unpublished data). In addition, as reported here, in 2011 in the Smolensk region, another set of imported animals imported from Germany was identified as carrying the BTV genome and anti-BTV antibodies during the quarantine period, although the laboratory results obtained before shipment into Russia were negative.

In this study, the BTV-14 strain isolated in the 2011 outbreak in the Smolensk region in Russia was characterized. Serotype/topotype analysis of the Russian BTV isolate relied on the comparison of Seg-2 and Seg-5 sequences and conventional serotyping. To determine the source of the infection and investigate its spread within the country, phylogenetic and evolutionary divergence data, as well as data from monitoring studies in the Smolensk region and neighboring areas, were analyzed.

## Materials and Methods

### Sample Collection

Serum and blood samples of 71 heifers from the farm “Smolensky Gallovei” in the Smolensk region of Russia (55°00'05.0“N, 34°28'49.1”E) were submitted in September 2011 to the Federal Research Center for Virology and Microbiology (Pokrov, Russia) for routine testing during quarantine in accordance with Russian legislation. The 71 young heifers of the Galloway breed were imported from Germany (Jurgen Greiner Ginsterweg) into the Smolensk region, following transport through Poland and Belarus. Before shipment, the animals were declared seronegative and PCR-negative for BTV (veterinary certificate 132 no. 0007170-0007171, dated 29.08.11).

The farm “Smolensky Gallovei” is typical in that it has a barn with open windows and animals are grazed daily in the field and rounded up twice a day for milking.

According to the BTV testing protocol, the animals were sampled for PCR and ELISA twice, at days 3 (05.09.2011) and 27 (29.09.2011) of the quarantine. Following the identification of infection, the quarantine was extended, and additional samples from the seropositive animals were collected on days 73, 104, 138, 189, and 207 (Table 1).

**Table 1:** Examination of the samples from the imported cattle for the presence of BTV genome and anti-BTV antibodies made during the quarantine (3–207 days) on the territory of Russia by real-time RT-PCR and ELISA.

**No animal**	**3 days**		**27 days**		**73 days**		**104 days**		**138 days**		**189 days**		**207 days**	
	**RT-PCR, Ct**	**ELISA**	**RT-PCR, Ct**	**ELISA**	**RT-PCR, Ct**	**ELISA**	**RT-PCR, Ct**	**ELISA**	**RT-PCR, Ct**	**ELISA**	**RT-PCR, Ct**	**ELISA**	**RT-PCR, Ct**	**ELISA**
75981	Neg	Neg	27.8	Pos	30.3	Pos	27.3	Pos	25.7	Pos	Neg	Pos	Neg	Pos
01051	Neg	Neg	24.9	Pos	27.9	Pos	23.8	Pos	24.2	Pos	Neg	Pos	Neg	Pos
94835	Neg	Neg	Neg	Pos	neg	Pos	Neg	Pos	Neg	Pos	Neg	Pos	Neg	Pos
01244	Neg	Neg	26.3	Pos	28.6	Pos	25.9	Pos	24.9	Pos	Neg	Pos	Neg	Pos
10055	Neg	Neg	25.6	Pos	30.0	Pos	26.7	Pos	25.7	Pos	Neg	Pos	Neg	Pos

Results are representative of three independent experiments.

*RT-PCR, real-time reverse transcription–PCR; Ct, cycle threshold; Pos, positive study result; Neg, negative study result*.

**Figure 1:**
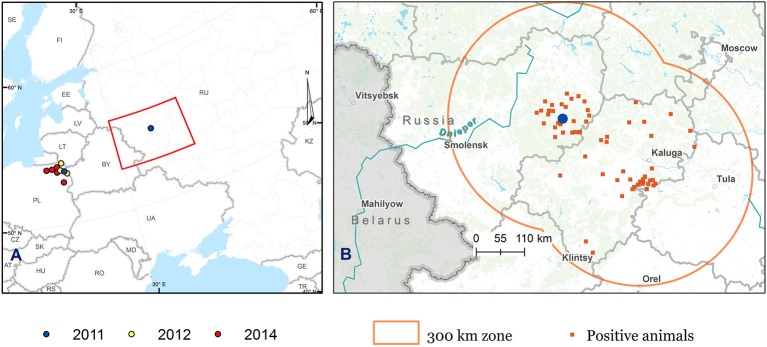
Map showing the distribution of BTV-14 in Poland and Russia. **(A)** Map of the European part of Russia and Eastern Europe. **(B)** Map of the European part of Russia showing the areas of monitoring survey. The blue circles show the location of the site of the first case of BTV-14 in the Smolensk region, Russia (the farm “Smolensky Gallovei”) and the location of Łowczyki village, the site of the first case of BTV in Poland, both in 2011. The areas of monitoring survey (Smolensk and Kaluga regions) are marked in orange. The orange squares refer to farms with BTV-infected animals identified in 2011 and 2012. The BTV-14 cases identified in Poland in 2012 are indicated by yellow circles, while the red circles indicate BTV cases detected in Poland in 2014.

As part of the study, in 2012, an additional 1623 serum and whole-blood samples were collected from clinically healthy cattle, sheep, and goats from different farms in the Kaluga and Smolensk regions within a radius of 300 km from the outbreak site (Figure 1).

### Virus Isolation

Blood was used for the first round of virus isolation in 10- to 12-day-old chicken embryos, according to the protocol described by Clavijo et al. (29). The inoculation of embryonating chicken eggs is more sensitive than cell culture, allowing detection of BTV in samples containing a low viral concentration (30). The blood (1/10 dilution) was intravenously inoculated into an embryo vein, incubated at 33.5 ± 0.5°C, and monitored daily for 7 days. Chicken embryos with pathological signs were further processed for downstream inoculation onto Vero cells, which were inoculated with filtered material in a T25 flask. Cytopathic effects (CPE) in the cell monolayers were used to detect the virus. The development of CPE was observed daily for up to 7 days post-inoculation. The virus was titrated by visualization of CPE in Vero and BHK-21/13 cell cultures. Titers were expressed as median tissue culture infectious dose (TCID50), according to the Reed–Muench method (31).

### Laboratory Diagnosis and Virus Identification

Laboratory diagnosis of infection was performed using enzyme-linked immunosorbent assay (ELISA) and reverse-transcription polymerase chain reaction (RT-PCR). Blood serum was tested for anti-VP7 antibodies by using an ID Screen Bluetongue Competition ELISA kit (IDVet, France) following the manufacturer's recommendations. The signal-to-noise ratio (S/N% = OD Sample/OD NC × 100) was calculated, and a sample with a ratio greater than or equal to 40% was considered negative, whereas a sample with a ratio less than 40% was considered positive. Then, serum samples with inconclusive results were retested.

Total RNA was extracted from the samples with TRIzol (Thermo Fisher Scientific, USA), precipitated with isopropanol, and washed with 75% ethanol, according to the manufacturer's protocol. Universal real-time RT-PCR directed against the non-structural protein 3 (NS3) fragment of the BTV genome was initially used to detect virus dsRNA in blood samples (32). The serotype 14 was identified using serotype-specific RT-PCR for genome segment 2 (Seg-2) (33). For both assays, the cutoff was set at a Ct-value of 38.

To exclude the other epidemiologically significant BTV serotypes in Russia, serotype-specific RT-PCR was used to test for BTV 1, 2, 4, 6, 8, 9, 11, and 16 (34).

To confirm the identified serotype, virus neutralization tests (VNT) against reference antisera to each of the 25 BTV serotypes (1–24, 26) were conducted. The VNT was performed using Vero and BHK-21/13 cell cultures as described by Koltsov (35).

### Sequencing and Phylogenetic Analysis

The serotype was confirmed by sequencing of the cDNA amplicons and by phylogenetic comparisons with previously characterized reference strains of each serotype.

Then, the samples that were identified as positive by universal real-time RT-PCR were subjected to VP2 (Seg-2, 2,922 nt) and NS1 (Seg-5, 1,772 nt) sequencing. Seg-2 and Seg-5 were chosen as serotype- and topotype-determining genes. Seg-2, together with Seg-5, encodes the proteins that define the BTV serotype, with the major contribution coming from VP2 (36). The complete gene sequences of VP2 and NS1 were determined by amplification of overlapping PCR products (three for segment 2 and two for segment 5) in the PCR assay, as previously described (35).

The cDNA was synthesized with primers specific to the ends of the BTV genome segments, using the SuperScript First-Strand Synthesis System for RT-PCR (Invitrogen, USA). The PCR was performed using specific primers and Quick-Load® Taq 2X Master Mix (NEB, USA). The PCR products were purified using a MinElute Gel Extraction Kit (Qiagen, USA). Sanger sequencing was used to assemble the complete sequences of Seg-2 and Seg-5 from overlapping PCR amplicons. The sequencing was conducted using the same primers in both directions.

SeqScape® Software for Mutation Profiling v. 2.5 (Applied Biosystems, USA) was used to obtain a consensus sequence. The VP2 and NS1 gene sequences were aligned using ClustalX (37). The full-length VP2 and NS1 sequence data of a BTV isolate were subjected to blast analysis using the NCBI BLAST tool (https://blast.ncbi.nlm.nih.gov/Blast.cgi) and were compared with sequences of commonly used reference strains and other strains represented in the GenBank (https://www.ncbi.nlm.nih.gov/genbank). Phylogenetic trees were constructed using the maximum likelihood method in which phylogenetic distances were estimated using Kimura's two-parameter model (K2P) (38) in the MEGA 7 program (39).

### Entomological Surveillance

Entomological investigations were conducted in the territory of the Smolensk region in 2012. *Culicoides* biting midges were collected in May from farms where BTV-14-infected animals were detected, and trapped using a CDC light trap with UV light (OVI traps) using a common technique. The trap was elevated 1.5 m above the ground near the cows and was operated from dusk (1800) to dawn (0800).

The collected midges were cooled, pooled, and morphologically identified into the Obsoletus and Pulicaris complexes using the keys described by Glukhova (40).

To identify the complexes at the species level, midge pools were tested using PCR assays based on mitochondrial cytochrome c oxidase subunit I gene amplification (41). RNA from insect pools (100 midges) was isolated using Trizol (Thermo Fisher Scientific, USA) according to the manufacturer's recommendations.

### Statistical Analyses

Statistical analyses were performed in the program VassarStats (http://vassarstats.net/).

## Results

Blood and serum samples of the 71 heifers from the same consignment in the Smolensk region were tested for the BTV genome using real-time RT-PCR and for anti-BTV antibodies using ELISA at days 3, 27, 73, 104, 138, 189, and 207 of quarantine. At day 3 of quarantine, all the animals were tested negative by real-time RT-PCR and ELISA (Table 1). At day 27, as well as at days 73, 104, and 138, antibodies were detected in five animals (nos. 01051, 01224, 75981, 94835, and 10055), four of which (the exception was no. 94835) were RT-PCR positive for the BTV genome at 138 days (Table 1).

At days 189 and 207, BTV RNA was no longer detected in the blood of the five animals, although anti-BTV antibodies were still detected.

### Virus Isolation

In the positive blood samples collected on day 27 of quarantine, the virus was isolated by inoculation into chicken embryos, followed by cultivation on Vero cells. After a 7-day incubation, the virus was harvested at 5.0 lg TCID50/cm^3^. A total of four BTV isolates were isolated.

The isolated BTV was capable of replication in different cell cultures (Vero, BHK-21/13, SK). The viral titer, depending on the cell line and the multiplicity of infection, ranged from 5.5 to 7.5 lg TCID50/cm^3^. Viral reproduction led to the destruction of the cell monolayer at 3–5 days post-infection.

### Virus Identification

The highest neutralization index (3.0 lg) was observed when the viruses isolated from the four animals (01051, 01224, 75981, and 10055) tested positive for reference BTV serotype 14 antiserum in the VNT. Simultaneously, the neutralization indexes when testing the virus samples with other reference sera did not exceed 0.75 lg, indicating that only the one serotype was present in the field.

The BTV-14 genome was detected in all blood samples of infected animals, as well as in the samples of infected chicken embryos and cell cultures that previously tested positive by universal real-time RT-PCR based on the NS3 protein. In the serotype-specific RT-PCR testing, the results were negative for BTV 1, 2, 4, 6, 8, 9, 11, and 16.

Thus, the virus isolated from the infected animals in the Smolensk region was identified as serotype 14 of BTV.

### Molecular Characterization

**Figure 2:**
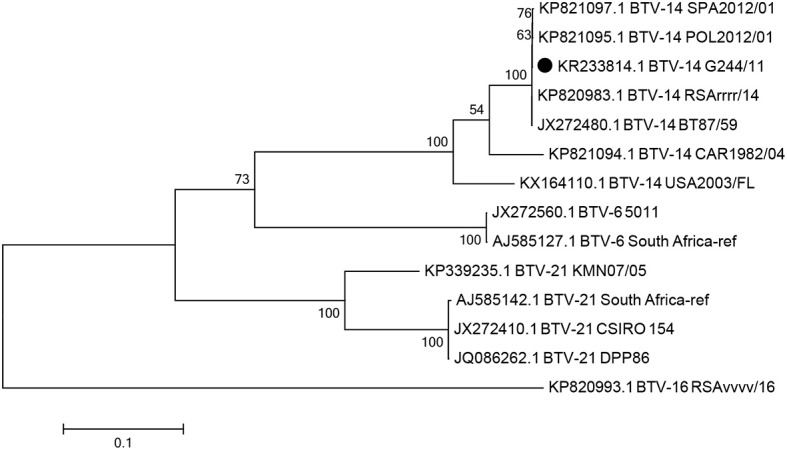
The maximum likelihood phylogenetic tree showing the relationships of the complete Seg-2 sequences (2,922 nt) of the G244/11 strain and other BTV strains of the Nucleotype C (BTV-21, BTV-6, BTV-14). The phylogenetic tree was constructed using MEGA 7. The percentage of replicated trees in which the associated taxa clustered together in the bootstrap test (500 replicates) with a percentage higher than 54 are shown next to the branches. The Russian BTV G244/11 strain isolated in the Smolensk region in 2011 is indicated by the black circle.

The VP2 and NS1 gene sequences of the G244/11 strain were deposited in GenBank under the accession numbers KR233814.1 and MN746787, respectively. The Seg-2 and Seg-5 nucleotide sequences of the four isolated viruses were all identical. In the phylogenetic analysis based on Seg-2 sequences of the G244/11 strain and previously characterized reference strains, the result of virus typing by VNT and serotype-specific real-time RT-PCR was confirmed. Thus, in the maximum likelihood tree, the G244/11 strain was grouped with BTV strains of serotype 14 with a bootstrap confidence value of 54–100% (Figure 2). Phylogenetic inference was used to place the G244/11 strain in a group comprising serotypes 6, 14, and 21 (Nucleotype C).

The sequence of the VP2 gene (Seg-2) had 99.93% nucleotide identity with that of the VP2 of the RSArrrr/BTV 14 (AJ585135) strain that was part of a polyvalent vaccine preparation, which included serotypes 1, 4, 6, 12, and 14 (Onderstepoort Biological Products, South Africa). The Seg-2 sequence was also similar to the sequences of European BTV-14 strains SPA2012/01 (isolated in Spain from animals imported from Lithuania) and POL2012/01 (isolated in Poland), with nucleotide identity of 99.86 and 99.90%, respectively.

The NS1 gene analysis showed that G244/11 belonged to the western topotype, sharing 100% nucleotide identity with RSArrrr/14 and BT87/59, both being BTV-14.

### Monitoring Survey

In 2011, BTV-14 was detected for the first time in the Russian Federation. As described previously, the virus was isolated only from cattle imported from Germany, during the quarantine period. To evaluate the incidence of BTV-14 among local livestock, local cattle, sheep, and goats grazing within a 300-km radius were sampled. To assess the prevalence of BTV infection, a serological and virological survey was conducted in local cattle, sheep, and goats from farms in the Smolensk and Kaluga regions between 2011 and 2012 (Figure 1).

A serological survey was conducted between 2011 and 2012 for specific antibodies against BTV using ELISA. The results for the seroprevalence of BT among animals in these regions are presented in Table 2. The seropositivity of cattle, sheep, and goats for BTV was 29.02% (471 of 1,623 animals, 95% CI = 26.83–31.31%). The highest seroprevalence was among ruminants in the Kaluga region (51.08%; 285 of 558 animals, 95% CI = 46.85–55.29%). Additionally, the BTV-14 genome was detected in 183 of the serologically positive animals (Ct values ranged from 26.1 to 37.6) by serotype-specific real-time RT-PCR and by universal real-time RT-PCR, whose results were 100% correlated (Table 2).

**Table 2:** Serological and virological investigation of local animals in the Smolensk and Kaluga regions during 2011–2012.

**Region**	**Year**	**Animals**	**Number of animals**	**Seroprevalence**	**Number of PCR-positive animals (Ct)**
Smolensk	2012	Cattle Sheep Goats	1065	17.46% (186/1065, CI = 15.25–19.9)	85 (Ct 26.1–37.6)
Kaluga	2012	Cattle SheepGoats	558	51.08% (285/558, CI = 46.85–55.29)	98 (Ct 26.8–37.7)
Total			1623	29.02% (471/1623, CI = 26.83–31.31)	183 (Ct 26.1–37.7)

The negative results of the serotype-specific RT-PCR tests for BTV 1, 2, 4, 6, 8, 9, 11, and 16 eliminated the other serotypes and indicated that only the BTV-14 serotype was actively circulating in the populations of local animals in the studied regions.

### Entomological Investigation

A total of 4,963 midges were collected, of which 366 (7.37%) belonged to the Obsoletus complex and 4,597 (92.63%) to the Pulicaris complex. Molecular analysis confirmed that the four species of the *C. pulicaris* complex (*C. pulicaris* s.s., *C. punctatus, C. impunctatus*, and *C. grisescens*) were present in the pools of biting midges.

Four pools of Obsoletus complex midges and 46 pools of Pulicaris complex midges were tested. Among them, only three pools of Pulicaris complex midges tested positive for BTV-14 RNA, with Ct values ranging from 31.3 to 33.9. The Obsoletus complex midges had no detectable BTV genome. When Seg-2 was sequenced, the sequences of the positive midge pools proved identical to those of the animals, indicating the same virus.

## Discussion

The key factors driving the incursions, transmission, and overwintering of BTV in Europe remain poorly understood (42). In particular, the unexpected incursions into new territories, of which the arrival of BTV-8 in Northern Europe is a good example (43), require elucidation.

The Russian territory bordering Europe experiences prolonged, cold winters followed by relatively short summers, which do not seem to favor the spread of BTV and make overwintering unlikely (44). Nevertheless, midges are abundant across the country (45). The first peaks of blood-sucking midge activity occur as early as late May, and secondary peaks, although less abundant, occur in July in Western Russia, dwindling dramatically in August (44, 46). However, the genetics and vectorial capacity of Russian midges remain to be poorly understood to conduct a risk analysis.

The serendipitous detection of BTV-14 in the cattle in the Smolensk region in 2011–2012 represents the first historical incidence of BTV in the European part of Russia in which antibodies and live viruses were identified in local livestock. The circulation of the live virus was confirmed by detection of BTV-14 in the neighboring Smolensk and Kaluga regions in 2012 (Figure 1, Table 2), as well as in Poland in 2012 and 2014 (47).

Sequencing of the VP2 (Seg-2) and NS1 (Seg-5) genes resulted in the Russian BTV-14 strain being grouped with a vaccine-like BTV-14 strain used in African countries (Figure 2). In addition to these findings, a related BTV-14 strain was also documented in Poland in animals near the Polish–Belarusian and Polish–Lithuanian borders in 2012 (47). Notably, Russian strain G244/11, strain SPA2012/01 detected in cattle in Spain imported from Lithuania, and Polish strain POL2012/01 shared approximately 99.9% identity based on the VP2 gene. However, the Russian strain carried two non-synonymous substitutions at position 1622 (C/T) and position 2427 (A/G) that were missing from the Polish and Lithuanian strains. This observation indicates the circulation of a live BTV-14 vaccine strain that spread asymptomatically and evolved in the field, likely in midges, before its detection in the Russian territory in 2011. The different BTV-14 strains were likely derived from a common source. However, when compared with the North American strains BTV-14, CAR1982/04, and USA2003/FL, Seq-2 of G244/11 shared 87.4 and 87.2% identity, respectively.

According to data obtained from Rosselhosnadsor (Russia), there was no evidence of infection in local animals before 2011 or after 2012 (http://fsvps.ru/fsvps/iac/rf/reports.html). However, 58 seropositive animals were identified in the Kaluga region in 2010 during monitoring studies in Russia (*n* = 39,113 during 2009–2010) (http://fsvps.ru/fsvps/iac/rf/reports.html). In blood serum samples (*n* = 1963) collected in 2011 from different regions of Poland, antibodies against BTV were detected in 494 animals. However, no virus or viral genome was detected (47). Currently, no information is available on the prevalence of the disease in past decades in Belarus. Thus, the initial source of infection remains unclear.

Notably, the animals did not present with any clinical signs and remained asymptomatic throughout the outbreak, which significantly limited understanding of BTV epidemiology in the moderate continental climate. The third day of quarantine occurred in early September 2011, whereas the 27th day (when the virus could still be recovered) occurred in late September. The average temperature at dusk between the dates was 7–13°C. This range comprised temperatures that include the minimum temperature or even outside the optimal range for flight activity of midges at this time of the year in Smolensk (44–46). In addition, little actual flight activity was recorded during this period. Thus, the entomological evidence does not support the hypothesis that the outbreak originated in Russia with the animals becoming infected during quarantine. Moreover, although the infectious virus was recovered in late September (day 27), for infection to occur, the midges had to be active and abundant, which was not the case in Smolensk in September 2011. Therefore, the most likely scenario is that the heifers were infected en route in Poland or Belorussia, which have a milder climate and where, from May to September, the highest midge activity is observed (47). The failure to detect the virus at day 3 of the quarantine may be attributable to the incubation period of the disease. In this study, BTV RNA could be detected for only 111 days (Table 1), although the maximum period for which RNA can be detected in infected animals is up to 160 days (48).

Due to this outbreak and the following report by Russia in 2011 (Ref. OIE: 11439, Report Date: 30/12/2011, Country: Russia), the attention of the World Organization for Animal Health (OIE)^1^ was drawn and BTV-14 became the focus of attention in Eastern Europe beginning in 2012 (47, 49). Although Russia was the first to report BTV-14, the report followed the import and testing of the consignment of heifers and therefore does not mean that the infection first appeared in Russia. The infection may have appeared elsewhere, and spread over a long distance via midges that are not bound by national borders.

Importantly, a live vaccine containing BTV-14 is not available on either the EU or the Russian market, which indeed suggests previous illegal use of a BTV-14-based vaccine. Although no evidence is available to support this contention, according to Nomikou et al. (49, 50), whole-genome sequencing data suggest that BTV-14 strains that have spread in Europe and Russia are reassortants containing genome segments derived from different reference vaccine strains originating from South Africa (49, 50). Nevertheless, although a possible vaccine incident is suggested as the source of the infection, consistent with the sequencing data presented herein, evidence of the illegal use of a live vaccine is lacking, as previously noted. Moreover, the use of a polyvalent vaccine preparation that included serotypes 1, 4, 6, 12, and 14 (Onderstepoort Biological Products, South Africa) does not seem logical, because the greatest threat to this area is associated with the BTV-8 serotype.

A possible alternative explanation for the present incidence is infection via BTV-contaminated fetal bovine serum, which is used as a semen extender for artificial insemination of cattle (51). Unfortunately, data on the extent of its use in the farming industry are not available. In addition, contaminated serum or other components may have been used to produce other live vaccines for cattle or small ruminants, which have already shown potential for contamination with BTV, as reported for a sheep-pox vaccine (16). Following vaccination, the contaminating BTV can escape and begin to circulate in the field. This route seems most plausible for the reported case; however, this hypothesis lacks evidence on what type of vaccine may have been contaminated.

Collectively, the literature indicates that blood-sucking insects drive BTV transmission across countries (8, 19). The reassortant nature of BTV-14 in Eastern Europe suggests that midges are most likely responsible for the BTV diversity (49, 52). Nevertheless, the vector species for the virus in Western Europe remains to be determined.

Entomological surveillance conducted in the Smolensk and Kaluga regions in 2012 demonstrated detection of the BTV-14 genome in midges of the *C. pulicaris* complex but not in those of the *C. obsoletus* complex. This study is the first to show that midges in Russia can potentially serve as a vector for BTV. However, this claim is made with the following caveats. In this study, midges were not sorted into nulliparous and engorged midges, and the focus was only on PCR positivity. Therefore, the results could indicate a positive blood meal rather than infected midge heads. This finding warrants further study to determine whether Russian midges can potentially be infected and subsequently transmit BTV. Another interesting observation was that Pulicaris complex midges dominated the catch, although Sprygin et al. (44) reported that Obsoletus complex midges are very abundant in the Smolensk region. In addition, although BTV RNA was not detected in Obsoletus complex midges, only four pools were tested.

Another limitation of the current study is that archived samples approximately 1 year before importation of the animals could not be accessed. This information would have provided insight into the actual prevalence of BTV-14 in the area during that period.

To obtain an update on the epidemiology of BTV in Russia, including serotype 14, an annual monitoring survey has been conducted. According to published reports by Rosselhosnadsor, no infected animals were detected from 2013 to 2019 in Russia (*n* = 217,286) (http://fsvps.ru/fsvps/iac/rf/reports.html).

In summary, this study describes the detection of asymptomatic BTV-14 infection in cattle in the Smolensk region of Russia in 2011. The identified BTV strain was genetically related to a vaccine BTV-14 strain, based on Seg-2 sequencing. However, the origin of the outbreak remains speculative. Although the possible mechanism of spread seems to be via vector bites because the collected midges tested positive for BTV dsRNA, this assumption merits further investigation. Elucidation of the mechanism involved would provide crucial insights into the circulation and overwintering of BTV in northern latitudes.

## Data Availability Statement

The datasets generated for this study can be found in the https://www.ncbi.nlm.nih.gov/nuccore/MN746787, https://www.ncbi.nlm.nih.gov/nuccore/KR233814.1.

## Ethics Statement

An ethical review was not required for this study, according to local and national legislation, because this work does not contain any experiments on animals. Blood and serum samples from cattle, sheep and goats were collected using standard procedures to avoid the suffering of animals and followed the guidelines of the State Veterinary Service of the Russian Federation. Farmers consented for the participation of their animals in this study.

## Author Contributions

ST and DK contributed to the conception and the design of the study. AK conducted field and laboratory work. AG participated in epidemiological data analysis. ST, AK, and GK performed laboratory data analysis. GK and AK wrote and reviewed the manuscript. All authors read and approved the final manuscript.

### Conflict of Interest

The authors declare that the research was conducted in the absence of any commercial or financial relationships that could be construed as a potential conflict of interest.

## Footnotes

^1^https://www.oie.int/fileadmin/Home/eng/Animal_Health_in_the_World/docs/pdf/Disease_cards/BLUETONGUE.pdf.
